# Patient Interaction Phenotypes With an Automated SMS Text Message–Based Program and Use of Acute Health Care Resources After Hospital Discharge: Observational Study

**DOI:** 10.2196/72875

**Published:** 2025-07-18

**Authors:** Klea Profka, Agnes Wang, Emily Schriver, Ashley Batugo, Anna U Morgan, Danielle Mowery, Eric Bressman

**Affiliations:** 1 Department of Medicine, Perelman School of Medicine, University of Pennsylvania, 423 Guardian Dr, Philadelphia, PA, 19104, United States, 1 2155732740; 2 Leonard Davis Institute of Health Economics, University of Pennsylvania, Philadelphia, PA, United States; 3 Center for Health Incentives and Behavioral Economics, University of Pennsylvania Health System, Philadelphia, PA, United States; 4 Department of Medical Ethics and Health Policy, University of Pennsylvania, Philadelphia, PA, United States; 5 School of Engineering & Applied Sciences, University of Pennsylvania, Philadelphia, PA, United States; 6 Institute for Biomedical Informatics, University of Pennsylvania, Philadelphia, PA, United States; 7 Penn Data and Analytics Center of Excellence, Penn Medicine, Philadelphia, PA, United States; 8 Department of Biostatistics, Epidemiology & Informatics, University of Pennsylvania, Philadelphia, PA, United States

**Keywords:** mHealth, behavioral phenotyping, clustering analysis, SMS text messaging, transitions of care, mobile health, mobile phone

## Abstract

**Background:**

Automated bidirectional SMS text messaging has emerged as a compelling strategy to facilitate communication between patients and the health system after hospital discharge. Understanding the unique ways in which patients interact with these messaging programs can inform future efforts to tailor their design to individual patient styles and needs.

**Objective:**

Our primary aim was to identify and characterize distinct patient interaction phenotypes with a postdischarge automated SMS text messaging program.

**Methods:**

This was a secondary analysis of data from a randomized controlled trial that tested a 30-day postdischarge automated SMS text messaging intervention. We analyzed SMS text messages and patterns of engagement among patients who received the intervention and responded to messages. We engineered features to describe patients’ engagement with and conformity to the program and used a k-means clustering approach to learn distinct interaction phenotypes among program participant subgroups. We also looked at the association between these interaction phenotypes and (1) patient demographics and clinical characteristics and (2) hospital revisit outcomes.

**Results:**

A total of 1731 patients engaged with the intervention, among which 1060 (61.2%) were female; the mean age was 65 (SD 16.1) years; 782 (45.2%) and 828 (47.8%) patients identified as Black and White, respectively; and 970 (56%) and 317 (18.3%) patients were insured by Medicare and Medicaid, respectively. Using k-means clustering, we observed four distinct subgroups representing patient interaction phenotypes: (1) a high engagement, high conformity group (enthusiasts, n=1029); (2) a low engagement, high conformity group (minimalists, n=515); (3) a low engagement, low conformity group (nonadapters, n=170); and (4) a high engagement with an intense level of need group (high needs responders, n=17). Differences were observed in demographic characteristics—including gender, race, and insurance type—and clinical outcomes across groups.

**Conclusions:**

For health systems looking to leverage an SMS text messaging approach to engage patients after discharge, this work offers two main takeaways: (1) not all patients interact with SMS text messaging equally, and some may require either additional guidance or a different medium altogether; and (2) the way in which patients interact with this type of program (in addition to the information they communicate through the program) may have added predictive signal toward adverse outcomes.

## Introduction

Automated, bidirectional SMS text messaging has emerged as a popular strategy to engage patients in various clinical contexts. It has been used to promote the uptake of preventive health measures, augment chronic disease management, and support transitions of care [1-4]. Across these use cases, it has shown both varying levels of uptake and engagement and mixed effectiveness with respect to targeted clinical outcomes. One central challenge has been identifying who will most likely benefit from an SMS text messaging–based approach. While most programs have deployed a one-size-fits-all approach, it is reasonable to assume patients differ in their communication preferences and the way they interact with SMS text messaging. The Technology Acceptance Model, for instance, posits that perceived usefulness and ease of use are drivers of individuals’ adoption of new health technologies, underscoring the importance of aligning text-based interventions with diverse patient needs and expectations [5-7].

Transitions of care after hospital discharge have been an especially compelling use case for automated SMS text messaging, given that many health systems already use some outreach strategy—typically call-based—to follow up with patients, often as part of an effort to prevent readmissions [8,9]. These approaches require a large investment of staff time and are still limited in their ability to reach patients or engage them longitudinally. Automated SMS text messages can significantly scale up touchpoints with patients, allow for asynchronous communication, and only require staff involvement when needs are identified.

We designed and tested a 30-day program leveraging automated SMS text messaging for primary care patients after discharge to increase patients’ access to their practice and facilitate timely interventions. An initial pilot study suggested an association with lower readmissions, but a larger randomized controlled trial did not replicate this finding [10,11]. Most patients responded to some of the program’s messages, but we observed a wide range of interaction styles and levels of engagement. This aligns with other work that has found differences in messaging engagement across demographic characteristics [12].

The aim of this study was to characterize these varied engagement styles more precisely through behavioral phenotyping. Through a process of engineering features to describe patients’ interaction styles and clustering on those features, we describe distinct interaction phenotypes and examine their association with other patient characteristics and clinical outcomes. This approach could lead to more nuanced, patient-centered strategies for designing and implementing future automated messaging programs.

## Methods

### Overview

**Figure 1.**
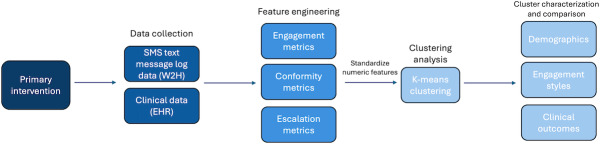
Workflow for data processing, from collection to phenotyping. EHR: electronic health record; W2H: Way to Health.

This was a secondary analysis of data from a randomized controlled trial that tested a 30-day automated SMS text messaging intervention among primary care patients after hospital discharge (ClinicalTrials.gov NCT05245773). We analyzed SMS text messages, patterns of engagement, and clinical data from patients enrolled in the intervention and who responded to any messages. Patients eligible for the original study were adults (aged 18 y or older) who received care in 30 primary care practices within the University of Pennsylvania Health System; discharged to home from an acute care hospital; and identified as medium to high risk at the time of discharge (using an Epic Systems Corporation developed and validated point score based on clinical information presented in prior literature and generally available in the electronic health record [EHR]; see Multimedia Appendix 1 for further details) [13,14]. The SMS text messaging program was built on Way to Health, a platform created with National Institutes of Health funding to provide automated technology infrastructure in support of clinical care and care delivery innovation research [15]. Figure 1 shows the workflow from primary data collection through feature engineering, clustering, and characterization of phenotypes.

### Original Study Design

Patients in the original study were randomized 1:1 to either usual transitional care (a single phone call from the practice within 2 business days of discharge) or usual care plus receipt of a 30-day automated SMS text messaging program. The study operated under a waiver of informed consent, though patients were free to opt out via text at any time. A total of 2352 patients were included in the intervention arm.

### Intervention

SMS text messages were sent out on a tapering schedule after discharge. A typical message asked, “Is there anything we can help you with today?” If they answered no, there was no further action. If they answered yes, a follow-up message asked them to further categorize their need (eg, “I need help with my medicines”). Patients were also told to reach out anytime outside of a scheduled check-in context by texting “Call.” Patients who reported a need through either of the methods above (an “escalation,” which was routed to an EHR in-basket) would receive a follow-up phone call from the practice within 1 business day. See the original study for a full schedule and script of all messages [11].

Our automated SMS text messaging program applied prespecified rules to incoming messages from patients. For instance, when patients were prompted with “Is there anything we can help you with today?” the software accepted only a narrow set of responses (Y, Yes, N, or No). Despite this, patients could respond as desired without a limit on character count. Messages containing text lacking a prespecified response triggered an outbound SMS text message to patients stating, “I don’t understand that response. Valid choices are: [prespecified choices].”

### Characterizing Interaction Phenotypes

#### Feature Engineering

We undertook a process of feature engineering to describe patients’ interaction with the program. This was limited to individuals who sent at least one message (otherwise, there was no interaction to characterize). Features fell into 2 broad categories: program conformity (how participants complied with the program’s requirements for SMS text messaging) and patient engagement (all other measures, which generally describe the frequency and speed at which patients sent in messages). These are defined in Table 1.

**Table 1.** Feature definitions.

Feature	Category	Definition
Response rate (%)	Engagement	Percentage of check-in messages to which patients replied
Average response time	Engagement	Average response time for check-in messages
Average word count	Conformity	Average word count per inbound message
Error rate (%)	Conformity	Percentage of inbound messages that triggered a system error message (either out of 1 d response time or response format does not match requirement)
Inbound message count (per day)	Engagement	Number of inbound messages divided by the number of days the patient stayed in the program before readmission
Proportion texting “Call” (%)	Engagement	The proportion of patients who requested a call outside of a scheduled check-in window
Need level	Engagement	The number of times patients had an escalation over the course of their time in the program, categorized as levels (0=no needs, 1=one need, and 2=two or more needs)
Need category	Engagement	There were 5 options for patients to categorize what they needed help with: symptoms, medicines, appointments, help at home, or miscellaneous

#### Clustering Approach

We applied a k-means clustering approach to identify subgroups associated with distinct patient interaction phenotypes. K-means clustering was chosen due to its ability to divide the data into nonoverlapping clusters, its scalability in working with large datasets, its effectiveness in handling continuous variables, and its interpretability. It allowed us to explore different values of k through established metrics to identify an optimal solution that captured clinically relevant subgroups. We considered hierarchical clustering but opted against this approach as it does not scale well with large datasets and does not inherently define a fixed number of clusters.

We first standardized the following features: error rate, inbound message count per day, average response time, check-in response rate, and average word count. Next, to determine the optimal number of clusters, we computed both the sum of squared errors and the silhouette score. For the sum of squared errors, we used the elbow method to identify the point where the slope leveled off. For the silhouette score, we prioritized values of k where the average silhouette score was maximized. We selected k=4 as it balanced the trade-offs suggested by both metrics, and it separated a unique subgroup that we deemed clinically important to isolate. We then characterized these 4 clusters by patient demographics and clinical outcomes.

#### Identifying Differences and Associations Between Interaction Phenotypes With Patient Characteristics and Clinical Outcomes

As part of the original study, we extracted information from the EHR related to patient demographics (age, self-reported gender, and race or ethnicity, and insurance), clinical characteristics (index hospital length of stay, Charlson Comorbidity Index, and LACE [Length of Stay, Acuity of Admission, Comorbidities, and Recent Emergency Department Use] score), and clinical outcomes (7-, 30-, and 60-day hospital revisits, encompassing either an emergency department visit or readmission). We describe these characteristics across the entire analytic cohort and by cluster. To test for differences in characteristics and clinical outcomes across clusters, we used the chi-square test for categorical variables and ANOVA testing for continuous variables. We report those characteristics and outcomes with statistical significance.

All analyses were conducted in Python (version 3.10.5; Python Software Foundation).

### Ethical Considerations

The original study was reviewed and approved by the University of Pennsylvania Institutional Review Board (IRB) (Protocol: 849348). The requirement for informed consent was waived for the original study with approval of the IRB; participants were free to opt out of the text messaging program at any time. No compensation was provided to participants.

As a secondary analysis, this study was reviewed and deemed exempt by the University of Pennsylvania Institutional Review Board (Protocol: 855553). The original IRB approval covered secondary analyses, and the waiver of consent similarly applied. All data were deidentified.

## Results

### Overview

A total of 1731 patients engaged with the messaging intervention and were included in the analysis, among which 1060 (61.2%) patients were female; the mean age was 64.8 (SD 16.1) years; 782 (45.2%) and 828 (47.8%) patients identified as Black and White, respectively; and 970 (56%) and 317 (18.3%) patients were insured by Medicare and Medicaid, respectively (Table 2). The mean hospital length of stay was 4.2 (SD 4.6) days, the Charlson Comorbidity Index was 4.7 (SD 2.9), and the LACE score was 66.7 (SD 13.8).

**Table 2.** Patient characteristics by interaction phenotype.

Characteristic		All (n=1731)	Enthusiasts (n=1029)	Minimalists (n=515)	Nonadapters (n=170)	High needs responders (n=17)	*P* value
Age (years) mean (SD)		64.8 (16.1)	65.2 (15.5)	62.6 (17.3)	68.9 (14.9)	65.1 (17.8)	<.001
Sex, n (%)							.14
Female		1060 (61.2)	634 (61.6)	323 (62.7)	91 (53.5)	12 (70.6)	
Male		671 (38.8)	395 (38.4)	192 (37.3)	79 (46.5)	5 (29.4)	
Race or ethnicity, n (%)							<.001
American Indian, Alaskan Native, Native Hawaiian, or Other Pacific Islander		7 (0.4)	4 (0.4)	0 (0)	3 (1.8)	0 (0)	
Asian		28 (1.6)	18 (1.7)	8 (1.6)	2 (1.2)	0 (0)	
Black or African American		782 (45.2)	389 (37.8)	304 (59)	85 (50)	4 (23.5)	
Hispanic		3 (0.2)	1 (0.1)	0 (0)	1 (0.6)	1 (5.9)	
White		828 (47.8)	570 (55.4)	170 (33)	77 (45.3)	11 (64.7)	
Other race		43 (2.5)	24 (2.3)	17 (3.3)	1 (0.6)	1 (5.9)	
Unknown		35 (2)	19 (1.9)	15 (2.9)	1 (0.6)	0 (0)	
Payer, n (%)							<.001
Commercial		364 (21)	240 (23.3)	89 (17.3)	33 (19.4)	2 (11.8)	
Medicaid		317 (18.3)	162 (15.7)	124 (24.1)	27 (15.9)	4 (23.5)	
Medicare		970 (56)	589 (57.2)	268 (52)	102 (60)	11 (64.7)	
Other		80 (4.6)	38 (3.7)	34 (6.6)	8 (4.7)	0 (0)	
Hospital LOS^a^, mean (SD)		4.2 (4.6)	4 (4.0)	4.2 (5.0)	4.9 (6.2)	3.8 (3.0)	.14
LACE^b^ score, mean (SD)		66.7 (13.8)	65.5 (14.4)	68.4 (13.2)	70.2 (10.3)	67.5 (12.4)	<.001
Charlson Comorbidity Index, mean (SD)		4.7 (2.9)	4.6 (2.9)	4.7 (2.9)	5 (2.8)	4.6 (3.8)	.43

a LOS: length of stay.

b LACE: Length of Stay, Acuity of Admission, Comorbidities, and Recent Emergency Department Use.

### Interaction Phenotypes

Applying the k-means clustering method to the full set of features, we observed 4 distinct clusters of patient interaction phenotypes. Measures for each feature, stratified by cluster, can be found in Table 3. The enthusiast (n=1029) was characterized by both high engagement (responding to 91.6% of messages, for instance) and high conformity (a 4.6% error rate). The minimalist (n=515) was characterized by low engagement (response rate of 35.2%) but high conformity (4.4% error rate). The nonadapters (n=170) were characterized by both low engagement (39.1% response rate) and conformity (40.8% error rate). Finally, the high-need responder (n=17) was characterized by high engagement (82.4% response rate), moderate conformity (18.5% error rate), and notably, a high rate of inbound messages (3.1 per d) and requests for help (64.7% requesting a call outside of a check-in window).

**Table 3.** Engagement measures by interaction phenotype.

Engagement measures		Enthusiasts	Minimalists	Nonadapters	High needs responders
Message response rate, %		91.6	35.2	39.1	82.4
Response time in minutes, mean (SD)		55.1 (65.6)	92.4 (124.9)	294.5 (440.2)	23.8 (29.8)
Word count, mean (SD)		1.7 (1.6)	1.6 (1.4)	4.6 (6.6)	2.0 (1.2)
Error rate, %		4.6	4.4	40.8	18.5
Inbound messages per day, mean (SD)		0.3 (0.2)	0.1 (0.1)	0.2 (0.2)	3.1 (1.1)
Proportion requesting a call, %		12.4	7.8	11.8	64.7
Need level, mean^a^		0.6	0.5	0.5	1
Need category (%)					
Symptoms		6.4	12.4	9.4	5.9
Medications		14.4	14.8	14.1	35.3
Appointments		8.7	8.5	10.0	0.0
Help at home		0.1	0.1	2.0	0.0
Miscellaneous		22.6	13.8	11.2	47.1
Outcome hospital revisits, n (%)					
7 day		44 (4.3)	13 (2.5)	7 (4.1)	6 (35.3)^b^
30 days		163 (15.8)^b^	100 (19.4)	34 (20.0)	9 (52.9)^b^
60 days		268 (26.0)^b^	161 (31.3)	50 (29.4)	10 (58.8)[Table-fn T3_FN2]

a Need level was a categorical representation of the number of needs a given participant had during their time in the program (0=no needs, 1=one need, and 2=two or more needs).

b Denotes statistical significance (*P*<.05).

### Differences in Patient Characteristics Across Interaction Phenotypes

In Table 2, we observed differences in most demographic characteristics across interaction phenotypes. Nonadapters were more likely to be older (mean age 68.9, SD 14.9) and male (n=79, 46.5%) than other groups. Enthusiasts were more likely to be White (n=570, 55.4%) and have commercial insurance (n=240, 23.3%) than other groups. Minimalists were more likely to be younger (mean age 62.7, SD 17.3) and Black (n=304, 59%) than other groups. There were no major differences in clinical characteristics across phenotypes.

### Association Between Interaction Phenotypes and Clinical Outcomes

We tested the association between the four interaction phenotypes and the clinical outcome of hospital revisits. In Multimedia Appendix 2, the high-needs responders had significantly higher rates of revisits at 7, 30, and 60 days (35.3%, 52.9%, and 58.8%, respectively). The enthusiasts were the lowest overall at 30 and 60 days (15.8% and 26%, respectively), while the minimalists and nonadapters were similar.

## Discussion

### Principal Findings

We identified 4 distinct patient interaction phenotypes—enthusiasts, minimalists, nonadapters, and high needs responders—with a postdischarge SMS text messaging program and observed differences in both patient characteristics and outcomes across these groups. Notably, there was an association between high-needs responders and enthusiastic interaction phenotypes and the outcome of hospital revisits.

As the use of mobile health (mHealth) has grown, so has interest in how patients engage with it. Indeed, most studies of mHealth interventions include some measure of user engagement, ranging from app logins to message response rates [16]; however, very few capture the heterogeneity of patient interaction styles. The notion of digital phenotyping, more generally (characterizing interaction with a range of digital health tools), is a nascent but growing field [17]. A similar study of a remote blood pressure management program also characterized engagement styles with automated SMS text messaging specifically, though, notably, this was a different context in both its use case (chronic disease management) and its population (patients who actively consented, vs this study which used a waiver of informed consent and may therefore be more generalizable) [18].

While it is not surprising that patients vary in both the degree to which they engage and their ability to grasp the rules of the system, most programs (including the one studied here) are designed as a one-size-fits-all approach [2]. The notion of just-in-time adaptive interventions has emerged in recent years—with mHealth being a prime use case—which aims to find the right intervention, for the right patient, at the right time [19-21]. This design challenge is related to the one explored here, and in some respects, it considers similar data about the patient. It rests on a slightly different premise about the patient, however, which is that in the course of an intervention, patients may require different strategies to keep them engaged, and we can learn over time how to adjust the timing and intensity of our approach to meet their needs at that particular moment [22].

Behavioral phenotyping asks more fixed questions about patients—for example, is this someone who engages with mHealth technology at all, and if so, how? [23] This is especially important in a population health context, such as ours, where patients are not being recruited for the program but rather automatically enrolled. While we found a high rate of overall engagement in our initial study (79.5% of participants responded to at least 1 message) [11], our analysis here found that this simple measure masks significant heterogeneity in sustained engagement and understanding of program rules. Indeed, as posited by the Technology Acceptance Model [5], perceptions of both usefulness and ease of use may explain why some patients engage intensively while others disengage, underscoring the need for tailored approaches that align more closely with individual patient preferences.

Capturing this information early in the course of an intervention can inform distinct approaches for these groups of patients. For instance, enthusiasts may not need additional intervention. Nonadapters may need extra attention, which could entail additional coaching to help guide their use of the program or a basic needs assessment that may suggest an entirely different medium better suited to their communication preferences. On the other hand, minimalists—who seem to understand the use of the medium (based on low error rates) but simply do not engage—may be best suited for just-in-time adaptive intervention strategies. Although high-needs responders constitute only ~1% of participants, they account for a disproportionate number of 30-day revisits. Flagging patients who generate multiple escalations or unusually frequent messages early in the program would let care managers focus on proactive calls, expedited follow-up visits, and pharmacist reviews on this small but high-risk subgroup.

This can have important implications for digital disparities, as well. In our original study, older adults appeared to engage at comparable rates to the general population, based on a simple measure of engagement; we found here, however, that nonadapters tended to be older, suggesting a need for additional support. We also found that enthusiasts were more likely to be White (n=570, 55.4%), while minimalists were more likely to be Black (n=304, 59%), suggesting the need for alternative strategies to narrow the engagement gap.

Future iterations of postdischarge outreach could layer these phenotypes onto a broader, multimodal ecosystem that matches patient needs and preferences. For instance, a smartphone app that centralizes text, video check-ins, medication reminders, and symptom tracking, or the use of wearable devices that can passively monitor indicators of early deterioration and trigger proactive outreach [24,25]. For patients without smartphones, interactive voice-response calls can be used [26]. An artificial intelligence–driven messaging layer could adjust cadence, channel (text vs voice), and language complexity in real time based on each patient’s engagement phenotype and predicted risk.

Finally, a novel finding of this work is the potential predictive value of users’ interaction styles. While mHealth programs typically rely on information communicated directly through the program (eg, the content of patients’ SMS text messages), our findings suggest that additional information may be gleaned from how patients interact with the program. We found, for instance, that a small cadre of users (high-needs responders) who had very intense communication and needs (several messages per day, high rate of requesting calls) returned to the hospital at a very high rate (52.9% with a 30-day revisit). Conversely, enthusiasts—who had a high response and low error rate—had the lowest 30-day revisit rate. Taken from this perspective, interaction phenotypes can be considered as another input into the overall risk assessment of patients and may help inform who needs additional transitional care support after discharge.

### Strengths and Limitations

The limitations of this study include that it is a retrospective analysis of a study from a single academic health center, which may limit generalizability to other settings, although it contained several practices and had a diverse patient population. Given the time-limited nature of the original intervention (30 d), there was a limited number of outbound messages, although enough to capture a dispersion of engagement patterns (and patients could text freely outside of the scheduled check-ins). The associations between phenotypes and outcomes were purely descriptive and did not adjust for other potential confounders. Because the high-needs responder group contained only 17 patients (~1% of the cohort), its estimates are inherently imprecise. Finally, while we applied a k-means clustering approach, traditional methods for selecting the optimal number of clusters (elbow method for sum of squared error plots, silhouette score plots) did not converge well on a single answer (3 or 4 both demonstrated arguable change in slope); however, 4 clusters was selected as it identified a distinct subgroup—high needs responders—that was noticeably different from the others. New features may suggest a different k.

This study also had several strengths. It is one of the only efforts to characterize patients’ interaction styles with automated texting. We had access to data from a large trial and were able to analyze a large number of participants. Finally, as noted, the study did not require informed consent, and patients were enrolled automatically if they met the inclusion criteria, making for a more generalizable patient sample.

### Conclusions

In this secondary analysis of a postdischarge automated SMS text messaging program, we observed variability in patient engagement. While the initial program may be a scalable template for patient follow-up, our findings suggest two key considerations for future iterations of this approach: (1) some patients may benefit from alternative communication strategies or additional guidance, and (2) patient engagement patterns with the program—in addition to the information they share directly—could offer predictive signals into adverse outcomes. Future research should explore integrating these insights into transitional care messaging programs and digital health programs, more generally, to improve patient engagement and outcomes.

## Supplementary material

10.2196/72875 Multimedia Appendix 1 Supplementary table and figures.

10.2196/72875 Multimedia Appendix 2 Association between interaction phenotype and revisit rates within 7, 30, and 60 days from discharge.

## Abbreviations

EHR: electronic health record
LACE: Length of Stay, Acuity of Admission, Comorbidities, and Recent Emergency Department Use
mHealth: mobile health
