# Supplementary material for: Patient Interaction Phenotypes With an Automated SMS Text Message–Based Program and Use of Acute Health Care Resources After Hospital Discharge: Observational Study
Source: J Med Internet Res. 2025 Jul 18;27:e72875. doi: 10.2196/72875 (PMC12296205; doi:10.2196/72875)

**Supplement to:** “Patient Interaction Phenotypes with an Automated Text Message-Based Program and Use of Acute Health Care Resources After Hospital Discharge.”

This appendix has been provided by the authors to give readers additional information about the work.

Patients included in the initial trial were identified as medium to high risk at the time of discharge using an Epic System Corporation point score.

The UPHS Risk Score is a tool used to assess a patient's risk of adverse health outcomes by assigning points based on various risk factors. Each risk factor is assigned a specific number of points, ranging from 1 to 3, depending on the severity or frequency of the condition. The score is calculated by adding the points across these categories, with a scores ranging from 0 to 15.

**Table S1.** Calculation of UPHS Risk Score

| **Risk Factor** | **1 Point** | **2 Points** | **3 Points** |
| --- | --- | --- | --- |
| Age | 65-84 | 85+ |  |
| Number of acute admissions in the past year | 1 admission | 2 admissions | 3+ admissions |
| Number of ED visits in the past year | 1 visit | 2 visits | 3+ visits |
| COPD, CHF, Diabetes, or Chronic Liver Disease | 1 point per diagnosis |  |  |
| Depression | Has diagnosis of depression |  |  |
| No PCP | Patient doesn’t have a PCP |  |  |
| Medicaid | Patient is on Medicaid |  |  |

**Figure S1. Sum of Squared Errors by Number of Clusters (Elbow Method)**


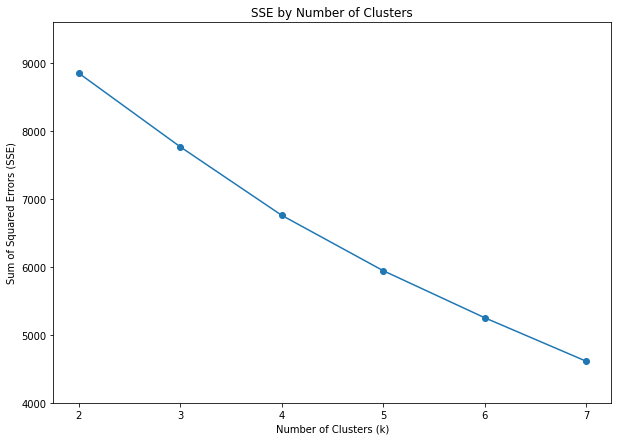


**Figure S2. Silhouette Score by Number of Clusters**


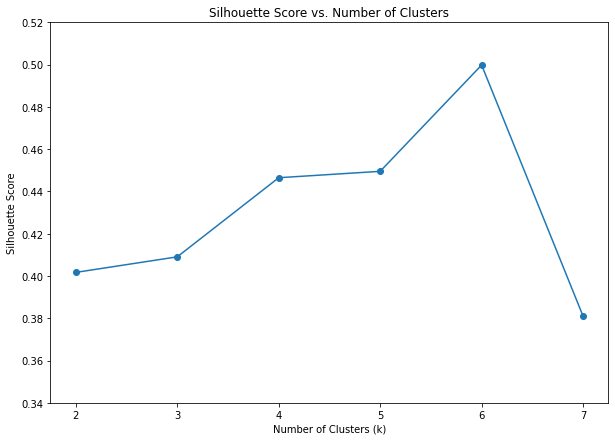

Supplement: Multimedia Appendix 1 [file jmir-v27-e72875-s001.docx]
