# Supplementary figures and images for: Patient Interaction Phenotypes With an Automated SMS Text Message–Based Program and Use of Acute Health Care Resources After Hospital Discharge: Observational Study
Source: J Med Internet Res. 2025 Jul 18;27:e72875. doi: 10.2196/72875 (PMC12296205; doi:10.2196/72875)

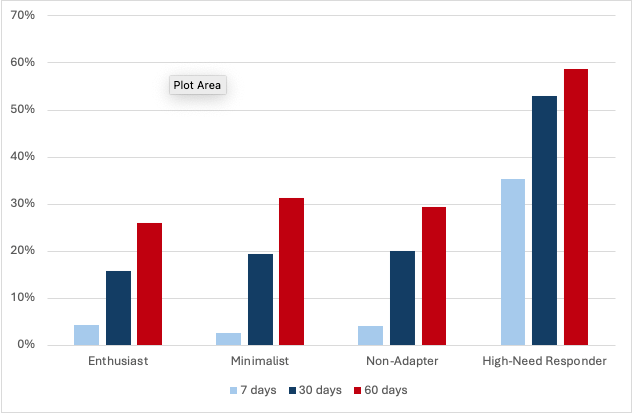

Supplement: Multimedia Appendix 2 [file jmir-v27-e72875-s002.docx]
